# Percutaneous Ultrasound-Guided Access to the Superior Ophthalmic Vein for Embolization of a Cavernous Sinus Dural Arteriovenous Fistula

**DOI:** 10.7759/cureus.8053

**Published:** 2020-05-11

**Authors:** Pham Hong Duc, Nguyen Thi Thai Hoa, Huynh Quang Huy, Anh Quoc Nguyen

**Affiliations:** 1 Radiology, Hanoi Medical University, Hanoi, VNM; 2 Oncology, Vietnam National Cancer Hospital, Hanoi, VNM; 3 Radiology, Pham Ngoc Thach University of Medicine, Ho Chi Minh, VNM; 4 Oculo-Facial Plastic and Cosmetic Surgery, Vietnam National Eye Hospital, Hanoi, VNM

**Keywords:** radiological intervention, cavernous sinus, arteriovenous fistula

## Abstract

The treatment of symptomatic cavernous sinus dural arteriovenous fistula, an unusually occluded cavernous sinus, is by a transvenous approach through the inferior petrosal sinus and superior ophthalmic vein. If these two modes of conventional transvenous access are not possible, surgical exposure and/or direct puncture access to the superior ophthalmic vein or cavernous sinus have been previously described. In a patient with progressive ophthalmological problems, the goal of treatment is to not only cure the ophthalmic symptoms but also to conserve or improve visual acuity, so treatment is needed as soon as possible. We report a 68-year-old woman suffering a Barrow type D cavernous sinus dural arteriovenous fistula. In this case, inadequate, inferior petrosal sinus embolization and lack of access for superior ophthalmic vein via a facial vein preceded percutaneous puncture under sonographic guidance of the superior ophthalmic vein. This permitted venous occlusion without complications and symptom-free for 11 months.

## Introduction

Spontaneous regression of cavernous sinus dural arteriovenous fistulas (CSDAVFs) is well recognized and explained by progressive restrictive changes in the affected cavernous sinus that contribute to the closure of the arteriovenous shunt [1]. Endovascular therapies have become the main therapeutic modality for symptomatic CSDAVFs [2]. In a patient with progressive ophthalmological symptoms related to CSDAVFs, rapid intervention may be necessary to prevent permanent visual loss [3]. Transarterial embolization for these fistulas through the meningeal branches of the external carotid artery and/or internal carotid artery has been limited to selected cases with the aim of palliative treatment but may contribute to alterations in venous drainage patterns resulting in a more aggressive natural history [1,4]. Transvenous embolization is therefore considered as the treatment modality of choice due to its low risk and a high likelihood of complete obliteration of the lesion. In most cases, the retrograde internal jugular vein approach through the inferior petrosal sinus (IPS) is successful. However, if the IPS is inaccessible due to reasons such as thrombosis or vessel tortuosity, the second common pathway is the superior ophthalmic vein (SOV) through the facial vein (FV). However, if the FV is not present, a direct puncture of the SOV or cavernous sinus (CS) has been reported [3,5,6]. Complications related to SOV puncture in the angiography suite, such as intra-orbital hemorrhage and thrombosis of SOV, have been reported, as in the present case [7,8]. However, these complications do not need management and may regress without ophthalmic problems.

## Case presentation

A 68-year-old woman presented with complaints of diplopia and decreased vision in the left eye and pulsatile tinnitus of left ear for several months, cluster headaches with high blood pressure despite having treated hypertension years earlier, and no traumatic history. She was consulted with and hospitalized at a local hospital. An eye drop preparation was prescribed, but the symptoms were still getting worse. On the last visit, she was referred to an ophthalmological hospital, and cephalic magnetic resonance imaging was performed, resulting in an enlarged ophthalmic vein (OV) suggesting a CSDAVF. She was referred to our hospital for treatment. Her visual acuities were 8/10 in the right eye and 3/10 in the left eye. Symptoms such as ptosis, conjunctival chemosis, and mild proptosis were observed. Orbital Doppler ultrasound revealed the enlargement of the SOV with pulsatile reverse flow on the left side.

**Figure 1:**
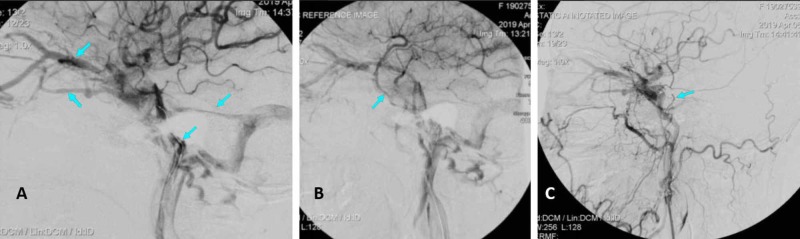
Left lateral view of internal carotid angiograms The arterial phase, early anterior (superior ophthalmic vein and inferior optic vein), and posterior (inferior petrosal sinus and superior petrosal sinus) drainage routes (A). The tardive phase, a variant of the superficial middle cerebral vein, drains via the foramen ovale into the pterygopalatine venous plexus (B). The left lateral view of common carotid angiograms of post coil embolization by transvenous inferior petrosal sinus showed complete occlusion of posterior drainage and persistent ophthalmic vein (OV) drainage, but no visible facial vein draining from the OV to the internal jugular vein (C).

The first procedure was performed under local anesthesia. A 4F guiding catheter was navigated into the involved carotid artery. Arteriograms showed meningeal branches of both internal and external carotid arteries were involved in the left side confirming a CSDAVF of Barrow classification type D. Both anterior drainage (superior ophthalmic vein and inferior ophthalmic vein) and posterior drainage (inferior petrosal sinus and superior petrosal sinus) were present; note the absence of superficial middle cerebral vein reflux form cavernous sinus (CS) (Figure 1). Treatment was performed with a 4F guiding catheter through the left internal jugular vein via the femoral vein to attempt to navigate a microcatheter into the CS via the IPS under angiographic roadmaps from the 4F guiding catheter in the carotid artery. This process was carried out several times using an ECHELON-10, 450-tip shape microcatheter with Avigo guidewire (ev3, Inc., Micro Therapeutics, Inc., Irvine, CA), but all failed because of septal stenosis between the IPS and CS. Based on this, we decided to use coiling to complete the occlusion of the partial posterior compartment of the CS and the IPS with a two-coil Axium (ev3, Inc.) with a size of 20 mm x 50 cm and 12 mm x 40 cm (Figure 1). Because of the absence of an FV, transvenous access retrograde to the OV was impossible. We decided to finish the procedure via a second intervention with the hope that the FV had opened while waiting for weeks.

**Figure 2:**
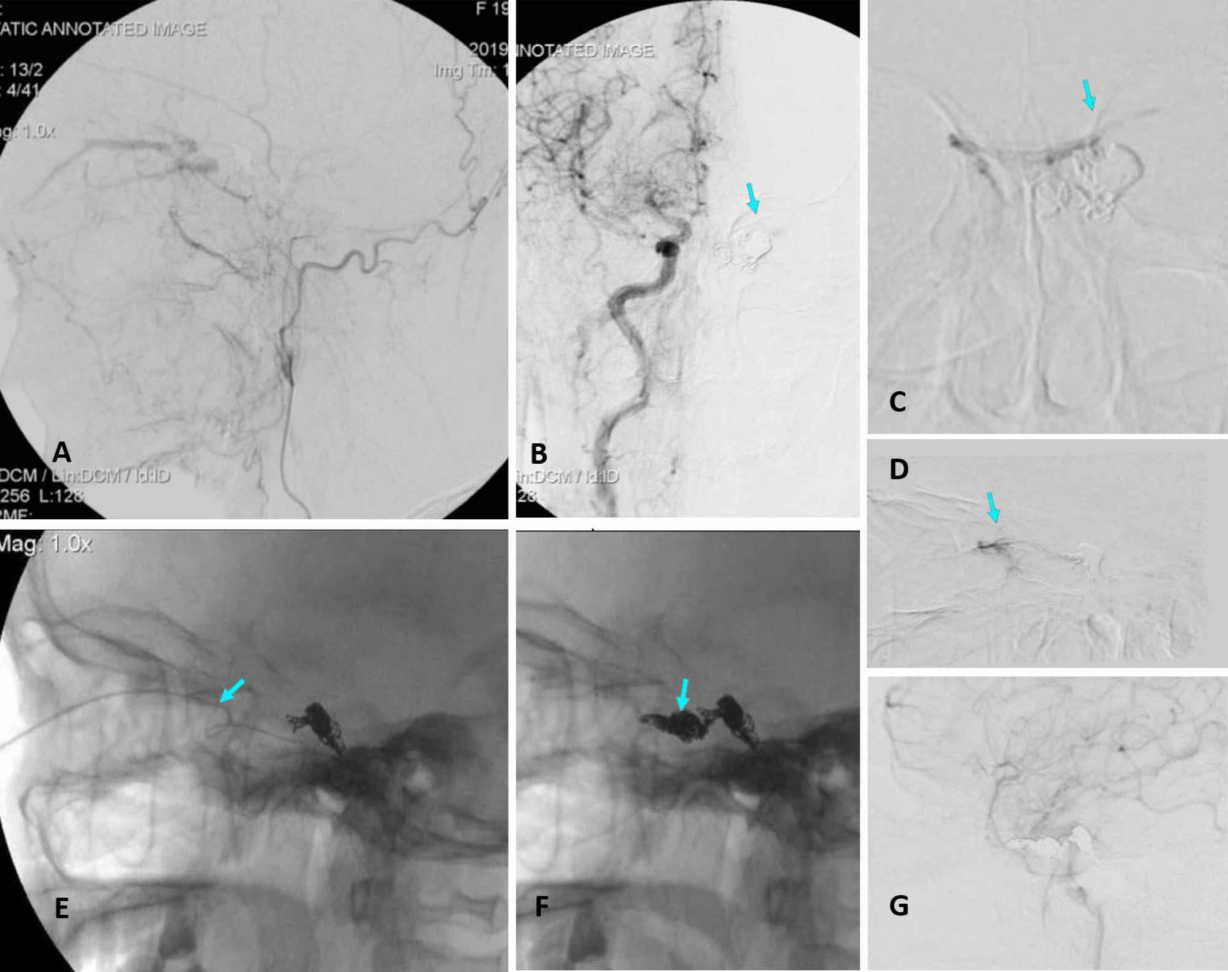
Left lateral view of external carotid angiograms showing no visualization of the facial vein (A, B) The anteroposterior (AP) view of the right internal carotid angiograms demonstrated a tiny left cavernous sinus (CS). AP (C) and lateral (D) views of the microcatheter tip in the left CS did not locate the ophthalmic vein (OV). (E, F) A microcatheter was introduced into the CS by direct puncture of the superior ophthalmic vein. (G) The deployed coil embolization in the anterior compartment of the CS and outflow of the superior ophthalmic vein leading to the complete occlusion of the cavernous sinus dural arteriovenous fistulas on the tardive phase of internal carotid angiograms.

After one month, the patient was hospitalized by appointment. Clinical examination found aggravation of proptosis and conjunctival hyperemia of the left eye after the first embolization of the IPS. The visual acuity of the right eye was stable at 8/10, while the left eye had decreased to almost invisible (1/10). The second procedure was performed under general anesthesia. Angiograms of left carotid arteries confirmed occlusion of the posterior drain veins but did not appear for the facial vein (Figure 2). We performed angiograms of the right carotid artery that detected low flow into the left CS via the inter-cavernous sinus (Figure 2). We tried to navigate the microcatheter from the right IPS to the left CS via the intercavernous vein, but superselective venograms showed no visualization of the SOV as an outflow tract (Figure 2). We decided to use direct percutaneous puncture of the SOV under ultrasound guidance.

**Figure 3:**
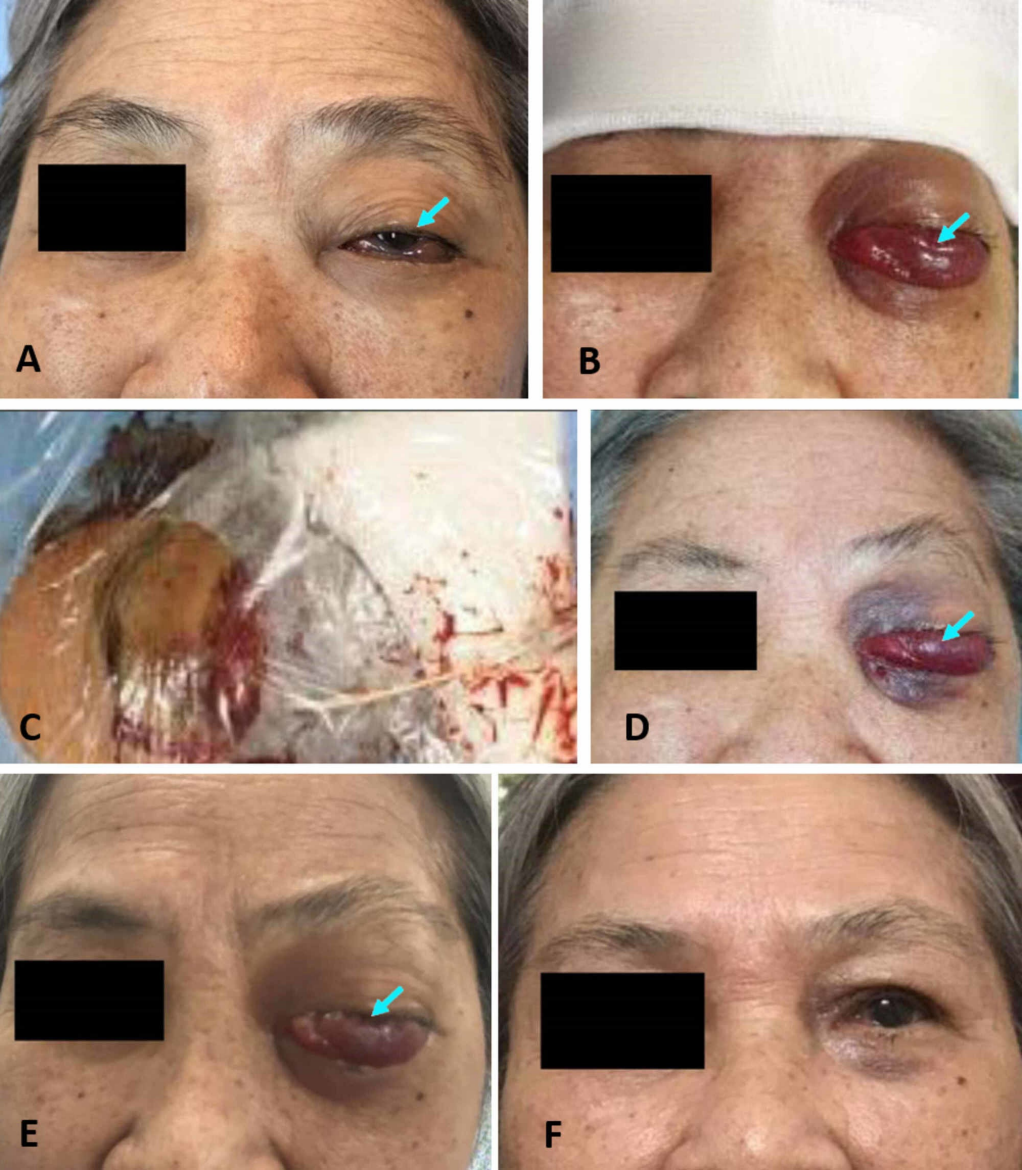
The affected eye before and after treatment Before the first intervention (A). Before the second intervention (B). After removal of the cannula connecting the perfusion Y hemostasis valve (C). Immediately after the intervention (D). Three days later (E) and three months later (F).

A 20G Terumo needle was used for the direct transorbital puncture. Doppler ultrasound was performed for determining the location of the SOV and the entry point for the needle. At the medial third of the inferior orbital rim, the needle was advanced under ultrasound guidance. When the needle was directed into the SOV, blood return was observed at the hub. The stylet was removed while a plastic cannula was inserted. This cannula was connected to a perfusion Y hemostasis valve (Figure 3). An ECHELON-10 microcatheter (ev3, Inc.) was advanced over an Asahi 0.008-inch micro-guidewire (Asahi Intecc Co., Ltd. Japan) through the cannula needle retrogradely into the superior ophthalmic vein and next to the cavernous sinus under fluoroscopic guidance on the lateral view (Figure 2). Superselective venograms were performed to confirm the correct position of the microcatheter tip within the CS. Subsequently, we pushed coils to slow down flow until control angiography confirmed the complete obliteration of the fistulas, with a total of five Axium coils (ev3, Inc.) of sizes 10 mm x 20 cm, 9 mm x 30 cm, 9 mm x 30 cm, 9 mm x 20 cm, and 5 mm x 20 mm used (Figure 2). The total heparin used in guiding catheter infusion was 5000 IU within six hours of the intervention.

The patient developed a medial-orbital hematoma immediately after treatment, but these symptoms recovered significantly during the subsequent three days after conservative treatment. After three months of clinical followed-up, her left vision still did not improve though no ophthalmic symptoms were observed (Figure 3). The patient has been symptom-free for 11 months.

## Discussion

Most CSDAVFs are indirect fistulas and fall under Barrow type D because there is always some supply from meningeal branches of both the internal and external carotid arteries [4]. With so many fistulas, this type usually produces a large flow into the cavernous sinus, causing severe clinical presentation and is difficult to treat by transarterial embolization. In order to explain the clinical symptoms and methods of intervention for this disease, venous drainage was mentioned, including posterior and/or anterior and/or cortical drainage routes [1,2,8]. In our patient case, arteriograms demonstrated the absence of cortical-pial venous reflux due to a variant of Sylvian vein drainage via the foramen ovale into the pterygopalatine venous plexus.

Symptomatic ophthalmic manifestations of CSDAVFs depend on veinous drainage patterns. With anterior venous drainage, increased venous pressure results in an increase in intraocular pressure leading to loss of vision, ocular pain, glaucoma, and retinal hemorrhage. Manifestations in the orbit include chemosis, exophthalmos, periorbital pain, and blephar edema. With posterior venous drainage, increased pressure in the cavernous sinus and vascular steal can result in cranial nerve deficits manifesting as ophthalmoplegia, diplopia, ptosis, or anisocoria [2]. In this present case, the initial symptoms presented both posterior and anterior drainage routes with mild ophthalmic symptoms. However, after one month from the occluded posterior route, ophthalmic symptoms were rapidly exacerbated and explained by an angiogram showing a marked anterior drainage route. However, it only involved the SOV, with no outlet from the SOV to the FV, and, in addition, had no Sylvian vein reflux.

Access to the cavernous sinus is considered a prerequisite for an attempt to cure an occlusion of CSDAVFs. In a case with only access by direct transorbital approach due to occlusion of all posterior routes and absence of FV, there are several methods used depending on the center and the experience of the interventionist. In the presence of dilated SOV, surgical exposure of the SOV through the upper eyelid margin for subsequent catheterization of the CS is a treatment option [9]. However, the surgical incision is relatively invasive and may cause cosmetic problems. A direct transorbital puncture has been reported as an alternative approach for accessing the CS after the failure of transvenous treatment [6]. Thanks to advancements in imaging technology, this approach is often successful. The safety is based on guidance from mapping digital subtraction angiography (DSA) or three-dimensional DSA and intraoperative cone-beam computed tomography images; this reduces risks of inadvertent puncture of vital structures [5,10]. This imaging technology can also help with direct puncture accessing the SOV and is a support tool for a microsurgical approach to the SOV [7,11,12].

The most recent retrospective study convincingly demonstrates that imaging-guided SOV access is a safe alternative approach for treatment in cases in which conventional transvenous approaches are not possible [7]. In the 20 cases of this report, real-time sonography-guided access was preferred as the first-line approach in 16 cases; only four cases used a biplane roadmap. One complication observed was a hematoma located based on the preseptal or postseptal SOV puncture. There were a total of six complications, of which sonography guidance found two preseptal and one postseptal hematoma. A preseptal hematoma does not require further management and will settle spontaneously within a week, as was seen in our case. A postseptal hematoma presents increased ophthalmic symptoms requiring emergency lateral canthotomy in the angiography suite and rapid normalization of vision after the procedure. Also, according to these authors, these guidance techniques achieved an angiographic cure in all patients, have an acceptable risk of associated complications, and provide a valuable and time-saving alternative route compared with direct surgical SOV. Another study found that retrobulbar hematoma due to rich vascular plexus bleeding will be gradually absorbed in several days after treatment and does not affect vision [10]. The complication of thrombosis of the SOV may occur immediately after puncture and, depending on the state of a drainage route, may cause postoperative non-arteritic ischemic optic neuropathy. To avoid this complication, puncture methods and heparinization should be considered after sufficiently investigating drainage routes [8].

Based on our initial experience, for success, this approach required an experienced interventional radiologist in Doppler ultrasound. The ability to access the SOV needs to be carefully evaluated to include the size and morphology (stenosis and loops) of the SOV, the entry point of the needle, and avoiding important anatomical structures such as the ocular globe and optic nerve.

## Conclusions

From this case, we learned that we need to intervene as soon as possible to avoid permanent vision loss. For the treatment of symptomatic CSDAVFs, in cases of inaccessible transvenous embolization, minimally invasive percutaneous ultrasound-guided technique can provide access to the SOV as an approach option. This technique was feasible, safe, and effective in our case.

## References

[1] Satomi J, Satoh K, Matsubara S, Nakajima N, Nagahiro S (2005). Angiographic changes in venous drainage of cavernous sinus dural arteriovenous fistulae after palliative transarterial embolization or observational management: a proposed stage classification. Neurosurgery.

[2] Thomas AJ, Chua M, Fusco M, Ogilvy CS, Tubbs RS, Harrigan MR, Griessenauer CJ (2015). Proposal of venous drainage-based classification system for carotid cavernous fistulae with validity assessment in a multicenter cohort. Neurosurgery.

[3] Benndorf G, Bender A, Campi A, Menneking H, Lanksch WR (2001). Treatment of a cavernous sinus dural arteriovenous fistula by deep orbital puncture of the superior ophthalmic vein. Neuroradiology.

[4] Barrow DL, Spector RH, Braun IF, Landman JA, Tindall SC, Tindall GT (1985). Classification and treatment of spontaneous carotid-cavernous sinus fistulas. J Neurosurg.

[5] Lyons LJ, Smith SA, Diaz O, Diaz H, Vickers A, Prospero C, Lee AG (2019). Percutaneous transorbital embolization of a carotid cavernous fistula. Proc (Bayl Univ Med Cent).

[6] Trivelato FP, Manzato LB, Filho PMM, Ulhôa AC, Vanzin JR, Abud DG, Rezende MTS (2018). Transorbital cavernous sinus direct puncture: alternative to treat dural arteriovenous fistula. Clin Neuroradiol.

[7] Heran MKS, Volders D, Haw C, Shewchuk JR (2019). Imaging-guided superior ophthalmic vein access for embolization of dural carotid cavernous fistulas: report of 20 cases and review of the literature. AJNR Am J Neuroradiol.

[8] Shinoda N, Mori M, Tamura S (2019). A patient with a cavernous sinus dural arteriovenous fistula in whom direct puncture of the superior ophthalmic vein led to rapidly progressing thrombosis and postoperative non-arteritic ischemic optic neuropathy: pathogenesis with respect to a drainage route. J Neuroendovasc Ther.

[9] Yeo MK, Ryu J, Choi SK, Kim EJ, Choi JM, Lee SH (2017). Direct superior ophthalmic vein access for dural arteriovenous fistula embolization. The Nerve.

[10] Xianli LV (2019). Direct trans-orbit puncture for embolization of the intra-orbital or cavernous arteriovenous fistula. Medocs Int.

[11] Fu ZY, Feng Y, Ma C, Chen JC, Krings T, Zhao WY (2019). Endovascular treatment of cavernous sinus dural arteriovenous fistulas via direct transorbital puncture using cone-beam computed tomography image guidance: report of 3 cases. World Neurosurg.

[12] Serratrice N, Baucher G, Reyre A, Brunel H, Fuentes S, Dufour H (2019). Management of two cavernous sinus dural arteriovenous fistulae by direct microsurgical approach and catheterization of the superior ophthalmic vein. Neurochirurgie.

